# The hidden economic and environmental costs of antimicrobial therapies: a call to action

**DOI:** 10.1017/ash.2024.496

**Published:** 2025-01-27

**Authors:** Mildred Nelson, Sarah B. Green, Sujit Suchindran, Lucy S. Witt

**Affiliations:** 1 Division of Internal Medicine, School of Medicine, Emory University, Atlanta, GA USA; 2 Division of Infectious Diseases, School of Medicine, Emory University, Atlanta, GA USA

## Abstract

The overuse and inappropriate use of antimicrobials have led to environmental waste and drug shortages. This challenges the ecological and economical sustainability of our healthcare system and worsens antimicrobial resistance.

Antimicrobial stewardship programs (ASP) commonly consider the cost of drug acquisition but may be failing to recognize the hidden costs of multi-dose intravenous regimens including additional nursing administration time, tubing and fluids, and potentially increased hospital length of stay. They also rarely consider the environmental impact of medical waste creation and disposal, which contributes to the global antimicrobial resistance crisis. These costs are harder to calculate but crucial to a comprehensive assessment of a medication’s total impact. In this invited commentary, we provide an example of a stewardship evaluation at our institution focused on changing from meropenem (MER) to ertapenem (ETP) for infections caused by extended-spectrum beta-lactamase producing organisms. We found that despite an increase in acquisition costs, changing from MER to ETP is associated with overall savings and decreased waste production. A secondary analysis suggests that stay length may also be improved with this substitution.

We present a holistic approach to antimicrobial stewardship that considers the total cost of an antimicrobial. By broadening their view to include hidden costs and secondary effects, ASPs can further demonstrate their value to the healthcare system, reduce resistance, and improve their environmental impact.

## Introduction

In the United States (U.S), nearly half of all hospitalized patients receive antimicrobials,^1^ many of which are unnecessary or overly broad.^2,3^ Inappropriate antimicrobial prescriptions lead to upwards of $65 million in excess healthcare costs in the U.S. for both adults and children.^4,5^ Antimicrobial stewardship programs (ASPs) are challenged with optimizing the spectrum of activity and dosage of antibiotics, while also weighing their pharmacoeconomic impact. Traditionally, this is limited to drug acquisition prices, but this may not encapsulate a medication’s total cost. In this commentary, we discuss the hidden costs and secondary economic and environmental effects of multi-administration intravenous (IV) antimicrobials. We provide an example of a more holistic cost evaluation undertaken by our ASP in order to highlight ways other institutions can adopt a more comprehensive approach.

## Hidden costs and secondary effects

**Figure 1.**
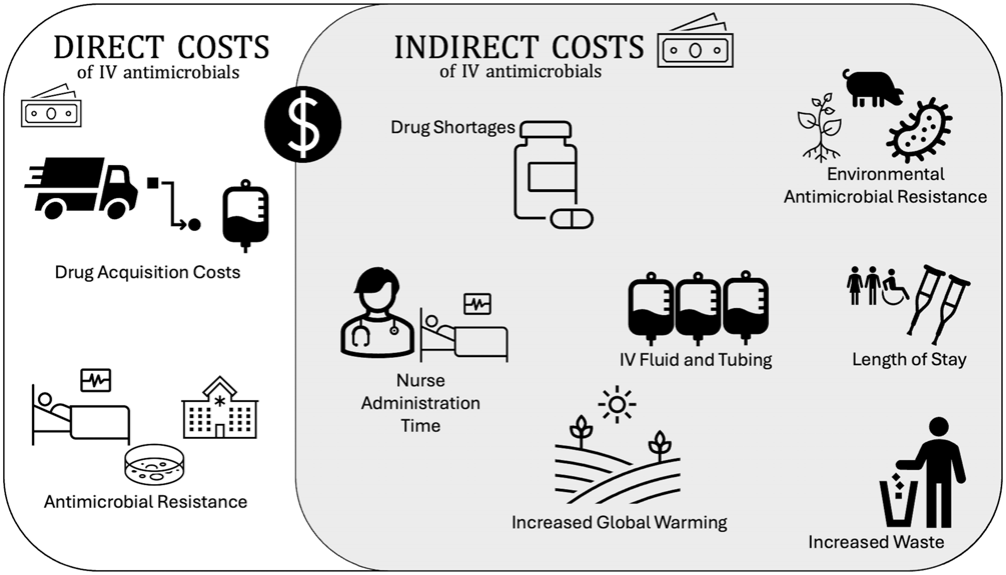
A visual representation highlighting the direct costs and indirect costs associated with IV antibiotic use.

Antimicrobials have indirect or “hidden” costs as well as secondary effects that are not traditionally assessed by ASPs (Fig. 1). These include costs associated with administration, such as tubing and carrier fluid – disposable materials are estimated to account for 13–113% of the total drug cost.^6^ Nursing time to administer drugs is similarly underappreciated. Studies have found it takes nurses twice as long to administer IV antimicrobials over infusion as compared to an IV bolus.^7^ Reducing nurse workload has been shown to shorten patient length of stay (LOS)^8^ and the Centers for Disease Control and Prevention cite reducing nursing time spent administering IV antibiotics as a cost-saving strategy to promote the change from IV to PO antimicrobials.^9^ Fewer IV infusions lead to fewer disposable materials used and reduced nurse time spent administering the antimicrobial.

Patients often transition to “destination” therapy on the day of discharge, a step required by many home health infusion companies. This transition can result in discarded doses of the original antibiotic leading to increased hospital costs and waste creation.^10^ The disposal of hazardous healthcare waste, including unused and expired medications, costs the U.S. approximately $1 billion annually,^11^ with antimicrobials being the largest contributor. Hospitals in the U.S. produce around 23 kg of pharmaceutical waste daily from antimicrobials, far surpassing the 1–3 kg generated by anesthesia or cardiovascular medications.^12^ This increased waste strains landfills, worsens global warming, and introduces antimicrobials into the environment contributing to resistance.^13,14^ Increased antimicrobials in the environment are believed to lead to antimicrobial resistance in livestock, water, and plants and have been hypothesized to contribute to colonization and even infection with resistant pathogens in humans.^15–19^

Patients who require multiple infusions per day may experience delays in diagnostics or have difficulty working with therapists leading to longer LOS, a hypothesis supported by a meta-analysis by Huang et al., which found that patients with infections from extended-spectrum beta-lactamase (ESBL)-producing *Enterobacterales* treated with IV ertapenem had a shorter LOS as compared to those treated with other carbapenems.^20^ As healthcare leaders remain focused on decreasing LOS, this argument may be particularly useful for ASPs working to prove their value.

Stewardship teams should routinely consider hidden costs and secondary effects when creating initiatives (Table 1). Taking these into account can provide evidence of ASPs’ value while reducing antimicrobial resistance and healthcare waste. Below, we describe an evaluation of our ASP conducted as part of regular workflow to holistically measure the cost and secondary effects of switching from extended infusion, multi-administration meropenem (MER) to once-a-day ertapenem (ETP).

**Table 1.** Direct, hidden costs and secondary effects of intravenous (IV) antimicrobials

Direct costs	Hidden costs and secondary effects		Stewardship interventions
Drug acquisition cost	IV Fluid Costs	Drug shortages	Prioritize IV drugs with daily or less frequent administration
Antimicrobial resistance in the patient microbiome and the healthcare system	Tubing costs	IV fluid shortages	Partner with pharmacy, nursing and/or case management to reduce unused antimicrobial doses
	Nursing administration time	Lack of progress toward discharge such as delays in imaging or working with therapy	Evaluate the effect on length of stay for stewardship interventions
	Equipment waste	Increased global warming	Partner with pharmacy to limit unnecessary administration equipment
	Antibiotic waste	Increased environmental antibiotic resistance	

## Our initiative

To evaluate the total cost of MER compared to ertapenem (ETP), we retrospectively identified all adult patients admitted to a single hospital (hospital A) with a bloodstream infection (BSI) due to *Escherichia coli* or *Klebsiella pneumoniae* with presumed ESBL activity (defined as phenotypic resistance to Ceftriaxone) over a six-month period. Patients were eligible if they received MER and either completed their course with MER or transitioned to ETP on discharge (N = 24). Patients who died during treatment, received high doses (2 g three times a day [TID]) MER, or transitioned to oral medications at discharge were not included. Hospital A is an academic, quaternary care facility with approximately 751 beds. Previously, hospital A had prioritized inpatient use of MER (administered as an extended infusion) over ETP due to the lower drug acquisition cost.

The estimated cost of materials including medications, fluids, and IV tubing was calculated using average wholesale pricing provided by the hospital pharmacy. The estimated cost of MER was $5.55 per 1-gm vial ($16.65 per day for patients with normal renal function) versus $23.85 per day per 1-gm vial of ETP. Each dose of MER and ETP is delivered with 100 mL of normal saline ($2.56 per bag). Intravenous tubing costs $4.16 per kit, and one kit is used per administration of medication. Total cost of medication was calculated based on the number of doses each patient received and was dependent on renal function. The total nursing cost was calculated using average time to administer an IV medication as calculated by Jenkins et al to be roughly 22 minutes and 5 seconds^21^; with an estimated cost of $15.94 nursing cost per dose administration. After taking these additional costs into consideration, the final cost of medication administration was $84.63 per day for MER TID compared to $46.51 per day for ETP 1 gm daily (Table 2). This resulted in an average of $217 of savings per patient if patients had switched from MER to ETP on day four of their seven-day treatment course, and an estimated $10,416 savings to the hospital over a year for just this small patient population. Furthermore, a change to ETP on day four would have resulted in at least 192 fewer IVs, bags of saline, and vials for disposal in six months at one hospital – a 66% reduction in waste.

**Table 2.** Estimated cost (in US dollars) of drug administration including drug, tubing, normal saline, and nursing administration cost

Product	Drug cost per day ($)	Tubing Cost per day ($)	Saline cost per day ($)	Nursing cost per day ($)	Total cost per day per patient ($)
Meropenem 1 GM TID	16.65	12.48	7.68	47.82	84.63
Meropenem 1 GM BID	11.10	8.32	5.12	31.88	56.42
Ertapenem 1 GM daily	23.85	4.16	2.56	15.94	46.51
Ertapenem 500 mg daily	23.85	4.16	1.25	15.94	45.20

Abbreviations: TID, three times a day; BID, twice a day.

**Figure 2.**
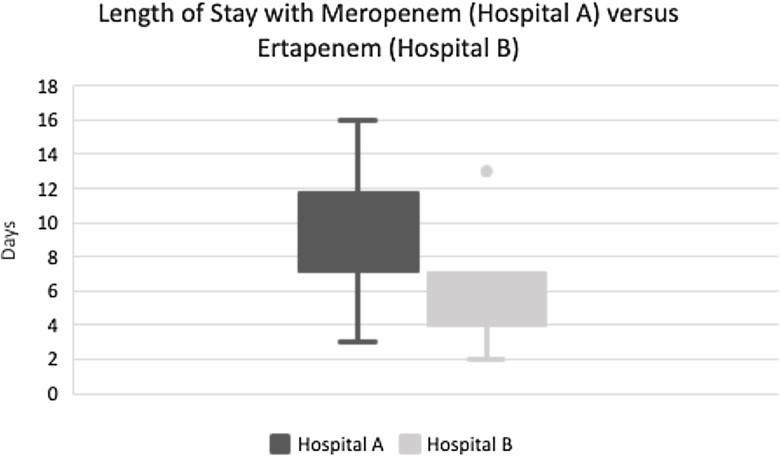
Median length of stay with range for Hospital A (using meropenem) versus Hospital B (using ertapenem).

To explore the potential effect of a switch from MER to ETP on LOS, we used a subset of the cohort of patients described above (hospital A) and compared them to a cohort of patients from a smaller academically affiliated community hospital (hospital B) admitted during the same period. Only patients with *E. coli* or *K. pneumoniae* ESBL BSI from a urinary source from each hospital were included. Hospital B includes obstetric care therefore pregnant patients were excluded to avoid potential confounding. Patients at hospital B must have been treated with ETP as their definitive therapy for inclusion. Prior to this evaluation, hospital B routinely utilized ETP for ESBL BSIs. We found that the mean LOS for patients with ESBL BSI from a urinary source at hospital A (treated with MER, n = 15) was 9.5 days, range 3–16, while patients at hospital B (treated with ETP, n = 6) had a mean LOS of 5.6 days, range 2–13 (student’s t-test*, P-value* 0.003) (Fig. 2). Although this evaluation does not account for all potential confounders, it does suggest that once-a-day medication administration may hasten hospital discharge.

In our analysis, converting IV MER, which requires multiple administrations per day, to once daily IV ETP demonstrated modest cost savings when considering total administration costs and a potential improvement in length of stay. Traditionally, ASPs may have viewed the change from MER to ETP as a mixed bag; ETP has an increased cost but narrower spectrum of activity, potentially providing reduced antimicrobial pressure on *Pseudomonas aeruginosa* in the hospital.^22^ Our widened review of an IV antimicrobial’s lifecycle also revealed the secondary effect of waste creation, suggesting both economic and environmental advantages to using medications like ETP that require fewer daily doses.

## Call to action: future directions

ASPs have traditionally focused on drug acquisition costs and antimicrobial resistance within their institution when implementing interventions. We demonstrate that data is available for a more comprehensive cost analysis, including medication, equipment, nursing, waste production, and length of stay. This broader perspective can enhance the value of ASPs and more accurately reflect the true cost of a medication. ASPs can play a key role in reducing healthcare expenditures and waste, aligning with the “One-Health” approach that links human, animal, and environmental health. Further research into the lifecycle costs of IV antibiotics and the impact of single-dose regimens on sustainability is essential to better protect healthcare resources and reduce antimicrobial resistance.
